# Supplementary material for: Are carbon emissions trading and green financial instruments synergistic? -Comprehensive quantitative research based on content analysis
Source: PLoS One. 2024 Mar 7;19(3):e0298601. doi: 10.1371/journal.pone.0298601 (PMC10919601; doi:10.1371/journal.pone.0298601)
Supplement: S1 Data — (ZIP) [file pone.0298601.s001.zip › Data/地级市Z_F工作报告.zip!/╜¡╦╒/─╧═¿╩╨/└┤╫╘╣1⁄2╓┌║┼í╛┬φ┐╦╩2╛▌═°í┐_2006.docx]

各位代表：

　　现在，我代表市人民政府向大会作工作报告。从今年开始，我们步入了"十一五"发展时期。根据《中共南通市委关于制定南通市国民经济和社会发展第十一个五年规划的建议》，市政府编制了《南通市国民经济和社会发展第十一个五年规划纲要（草案）》，请各位代表连同政府工作报告一并审议，并请市政协各位委员和其他列席人员提出意见。

　　一、2005年工作情况和"十五"主要成就

　　过去的一年，在省委、省政府和市委的正确领导下，全市上下认真落实科学发展观，开拓创新，扎实工作，市十二届人大三次会议确定的各项目标任务全面完成。

　　（一）经济增长速度全省领先，发展质态进一步优化。全市实现地区生产总值1472.08亿元，按可比价计算增长15.4%，增幅居全省首位。完成财政总收入171.19亿元，增长26.3%；地方一般预算收入71.99亿元，增长26.7%，高于全省平均增幅0.3个百分点。农业实现增加值162.19亿元，增长3%；工业实现增加值672.66亿元，增长19.6%，增幅居全省第二；建筑业实现增加值151.33亿元，增长17%；服务业实现增加值485.9亿元，增长14.4%。县域经济加速发展，在全市地区生产总值中的占比较上年提高1个百分点，南三县（市）保持良好发展态势，北三县（市）工业增加值、注册外资实际到帐等多项经济指标增幅高于全市平均水平。发展质态继续提升。规模以上工业实现利税增长36.4%，高于产值增幅13.6个百分点；工业经济效益综合指数达187%，为历史最好水平。高新技术产业产值比计划提前两年实现翻两番的目标，在规模以上工业产值中的占比较上年提高3.1个百分点。单位生产总值能耗预计为0.95吨标准煤，低于全国、全省平均水平。环境保护工作继续位于"全国污染控制较好城市"前列。

　　（二）江海联动开发取得重大突破，重大项目建设力度进一步加大。江海联动开发20项重点工作任务基本完成。沿江开发向纵深推进。现代纺织服装、船舶制造与配套、电子信息、精细化工、机械制造等优势特色产业发展态势强劲。有效盘活深水岸线资源，建成万吨级以上泊位5个，其中5万吨级以上泊位3个；全市港口货物吞吐量达8326.9万吨，增长15.4%。各口岸查验单位整体联动，"大通关"功能进一步完善。沿海开发实质性启动。洋口港锚地成功进行加载作业，临港工业区一期围堰工程完成，陆岛跨海通道和洋口大道开工建设；启东滨海工业集中区开工建设，吕四港大唐电厂一期围堰吹填工程竣工，两个5万吨级泊位岸线使用获交通部批准。一批重大产业及基础设施项目进展顺利。全市实施重大项目101个，比上年增加30个；项目单体规模超过9亿元，比上年增加1亿元。江苏LNG项目获国家发改委批准开展前期工作，中远川崎二期扩建、熔盛造船等48个重点项目开工建设，醋纤四期扩建工程丝束部分、天生港电厂技改工程等44个重点项目相继完工。苏通大桥顺利转入主桥索塔和上部结构施工，北接线高速路基桥梁贯通；崇启大桥完成工程可行性研究报告；皋张汽渡通渡。沿海高速南通段建成通车，沿江高等级公路全线开工。铁路"一线三中心"建设方案开始实施，沪通铁路着手开展建设前期工作。通过继续猛攻有效投入，完成全社会固定资产投资815.26亿元，增长34.7%；完成规模以上投资672.08亿元，占全社会固定资产投资比重超过80%，增幅居全省第一；完成工业投入617.72亿元，增长54.3%，总量列全省第三。

　　（三）开放型经济与民营经济比翼齐飞，发展活力进一步增强。全年新批协议外资突破50亿美元，同比增长34.2%；新增工商注册登记外资31.1亿美元，列全省第二；注册外资实际到帐17.48亿美元，列全省第三，跻身全国大中城市利用外资15强行列。利用外资质量进一步提高，新批总投资超千万美元的大项目198个，比上年增加49个；服务业实际利用外资增长84%。完成进出口总额85.2亿美元，增长25.4%。对外劳务完成营业额6.62亿美元，增长42.7%。全民创业势头强劲，私营企业、个体工商户新增数等六项指标列全省第一，民营经济增加值占全市地区生产总值的比重达43.1%，比上年提高2个百分点。先后在珠三角、长三角和浙东等地举行大型民资招商活动，正式签约项目287个，当年开工172个，总投资86亿元。争创"名企·名品·名人"工程取得成效，11家企业入围全省民营企业销售收入50强；新增中国驰名商标1个、中国名牌产品5个。以民营为主体的建筑业主要经济指标继续保持全省第一，新增特级资质企业3家，占全省新增数的四分之三，8家企业跻身全省建筑业综合实力20强，获全省建筑强市称号。国企改革继续深化，国有资产管理体制进一步完善。市属社会公益型事业单位改革全面启动，首批16家改革基本到位，第二批187家正在推进。新增上市公司1家，2家上市企业完成股权分置改革。乡镇综合配套改革试点工作初见成效。各类行业协会、商会进一步发展。接轨上海工作取得新成效，与长三角城市在港口、产业、科技、旅游等方面的合作得到加强。

　　（四）"三农"工作扎实推进，农村面貌进一步改善。按照城乡统筹的要求，抓好"三农"工作。在稳定粮食综合生产能力的前提下，农业结构不断优化，瓜果蔬菜、花卉苗木、家禽养殖、海洋渔业等主导产业优势更加突出。农业产业化进程加快，项目农业建设取得新成效，高效农业规模化水平进一步提高，四分之一的耕地亩产出超过3000元，市级以上龙头企业实现销售收入超百亿元，获国家认证的无公害农产品435个、绿色食品129个、有机食品14个，建成"四有"农民专业合作组织57个。通过加强技能培训和劳务中介服务，新转移农村劳动力6.26万人。加大财政支农力度，"一免三补"政策全面落实。"民富、村美、风气好"的新农村建设开始起步。高度重视村镇规划编制工作，稳步推进农民集居区建设；以农村环境整治为重点的"百村示范、千村整治"工程进展顺利，小康示范村建设起步良好；新建和改造农村公路里程是历史上最多的一年。

　　（五）城市建设保持大推进态势，城市知名度和影响力进一步扩大。以"五城同创"和举办"五大活动"为推动，全面提升中心城市建设和管理水平。中心城市建设强势推进，发展框架进一步拉开，通宁大道等18项城建重点工程相继完工，崇川路东延等15项跨年度重点工程完成年度任务，新增道路面积212万平方米。新区建设加速推进，体育会展中心建设进展顺利，中央商务区开工建设。积极推进商品房规模开发，市区商品房竣工面积163.25万平方米，比上年净增66.57万平方米；依法做好拆迁工作，城市房屋拆迁量比上年增加45.28万平方米。城市公用设施建设力度加大，市区污水日处理能力达到15.5万吨，污水处理率达73%；新增公交站点82个；电话号码升至8位。重点实施城市环境整治"3410"工程和"精品濠河"工程，市区10个重要节点环境明显改观，濠河亮化工程获全国市政工程"金杯奖"；新增绿地180公顷，绿化覆盖率达41.5%；整治河道23条；着力推进"五小"行业整治；启动姚港化工区三类企业搬迁工程。创建国家环保模范城市通过验收；创建国家园林城市通过省级考核验收，荣获"江苏省园林城市"称号，并获得省第五届园艺博览会举办权；创建国家卫生城市通过省级验收和复核。成功举办第二届世界大城市带发展高层论坛、2005中国南通港口经济洽谈会、南通博物苑一百年暨中国博物馆事业发展百年庆典、2005全国航空运动会、长三角城市经济协调会第六次会议，城市形象进一步提升。

　　（六）各项社会事业加快发展，精神文明和民主法制建设进一步加强。科技工作成果显着，市及各县（市）区均通过国家科技进步考核，市及海门、通州、启东、海安4县（市）获全国科技进步先进市（县）称号。列入国家科技成果重点推广计划数、省科技进步奖获奖数、省农业科技攻关项目数等列全省第一；新增国家级高新技术企业11家、重点新产品31项。人才人事工作跻身全国先进行列。教育现代化工程稳步推进，"六有"和"校校通"工程全面完成，58%的乡镇成为省级教育现代化乡镇；基础教育保持优势，我市素质教育、未成年人思想道德建设成为全国典型，高考生均总分等十项主要指标继续保持全省第一，职业教育、高等教育、成人教育进一步发展。加快发展文化事业，建成南通博物苑新馆、城市博物馆等一批文化设施，环濠河博物馆群基本形成。广播电视事业发展加快，当年有线电视用户发展数位居全省第一。成功承办全国十运会击剑比赛，南通籍运动员在十运会上获得14项次金牌，积极筹备省十六运，全民健身工程向农村延伸。加大公共卫生事业建设力度，健全疾病预防控制体系，重大疾病防治工作取得新进展；加强医院管理，医疗服务水平有了新的提高；综合卫生监督进一步加强，乡镇卫生所规范化建设全面推进。计划生育率达99.6%。广泛开展"南通精神"大讨论活动，群众性精神文明创建活动成效显着。法治南通建设向纵深推进。制定加快法治政府和法治南通建设实施意见，出台相关配套措施，在全省较早建立行政首长出庭应诉制度。切实帮助群众解决实际问题，信访总量明显下降。平安南通建设和全省最安全城市创建工作取得新成效，社会矛盾纠纷大调解机制和社会治安大防控体系进一步完善，市及通州市分别获得全国社会治安综合治理优秀市和先进县（市）称号。南通刑警被国务院授予"特别能战斗刑警队"称号，我市进入全国首批"科技强警示范城市"行列。安全生产工作得到强化。广泛开展双拥共建活动，国防教育、征兵、民兵预备役、人民防空、国家安全工作均取得新成绩。统计、外事、侨务、气象、民族、宗教、防震、无线电管理、档案、保密、地方志等工作进一步加强。

　　（七）城乡居民生活质量继续改善，社会和谐程度进一步提高。全年新增城镇就业5.5万人，年末城镇登记失业率为3.4%。社会保障覆盖面继续扩大，市区养老保险基金收支基本平衡。农民人均纯收入5501元，增长11.6%；市区居民人均可支配收入12384元，增长13.2%。居民消费价格指数为101.2%。被拆迁群众和被征地农民补偿款全部发放到位，基本生活得到保障。老龄事业和产业得到发展。妇女、儿童、青少年、残疾人、关心下一代、见义勇为、红十字和慈善工作取得新成绩。认真履行承诺，10件14项实事项目完成情况良好。其中，提前或超额完成的10项：(1)全年实现5.7万名城镇失业人员就业，超过计划的62.9%；(2)全市新型农村合作医疗覆盖面达87%，高出计划7个百分点；(3)全市建成社区卫生服务中心117个、社区卫生服务站1828个，覆盖率达95.7%；(4)完成农村改水攻坚任务，农村自来水普及率达98%，高出计划3个百分点；(5)市区新建低价位商品房81.17万平方米，超过计划的35.3%；(6)改造农村危桥360座；新建和改造农村公路2248.71公里，超过计划的72.9%；(7)疏浚县级河道25条、乡级河道154条；(8)城市低保提标10%，农村低保提标10%左右；(9)市区安排100万元用于送温暖工程，同时为符合条件的定补（保养）职工增发生活补助金616万元；(10)全面推行农村部分计划生育家庭奖励扶助制度，对44889名农村居民按标准发放了奖励扶助金。按计划如期完成的3项：(1)市污水处理厂二期、江海港区污水处理厂建设工程竣工，观音山污水处理厂一期工程开始建设；(2)市区端平桥、新桥等4个农贸市场改建工程按计划完成；(3)在落实企业离退休省级以上劳模待遇的同时，提高了企业离退休市级劳模的待遇。已完成年度工作计划，需继续跨年度实施的1项：西北片区域供水工程、启海区域供水工程正式开工建设。

　　（八）政府自身建设得到加强，行政效能进一步提升。2005年是规划编制年，也是项目突破年。围绕科学规划、项目推进等重大问题，有针对性地组织开展89个专项和系列调研，80%的调研成果得到转化。集思广益，精心编制《南通市国民经济和社会发展第十一个五年规划纲要（草案）》和51项专项发展规划；制定了《南通市生产力（工业产业）空间布局与工业集中区规划》等3个空间布局规划，基本完成市级土地利用总体规划大纲编制工作；启动城市总体规划修编，完成城市公共交通等6个专项规划及市区22平方公里控制性详规等12个修建性详规的编制；编制完成125个乡镇布局规划和13个市级小康示范村规划。进一步加强政府目标责任管理，健全政府常务会议督查制度，强化重大项目推进制度和领导挂钩、督查制度，化解土地、电力、资金等要素瓶颈制约，完善经济工作推进机制，112项重点工作完成情况良好。注重政府法制建设，行政执法监督得到加强。全面推进政务公开，政府采购、工程招投标全面实行阳光操作，进一步加强市、县两级行政审批中心建设，完善行政效能监察机制，机关行政效率明显提高。自觉接受市人大及其常委会的法律监督、工作监督和市政协的民主监督，认真执行人大及其常委会决议，支持政协履行职能，切实抓好人大议案和人大代表建议、批评、意见以及政协委员提案的办理工作，市人大代表和政协委员满意基本满意率分别达95.9%、98.8%。主动听取、充分吸纳市人大、市政协意见和社会各界的意见和建议，提高了政府科学决策、民主决策水平。认真落实党风廉政建设责任制，领导干部廉洁从政自觉性进一步提高，审计监督进一步加强。

　　各位代表，过去的一年为"十五"发展划上了圆满的句号。五年来，全市人民紧紧围绕争当全省江北"两个率先"排头兵的目标，按照市委确定的"依托江海、崛起苏中、融入苏南、接轨上海、走向世界、全面小康"的总体思路，以跨越发展为主题，解放思想、奋力拼搏、真抓实干、争创一流，经济社会发展取得了令人瞩目的成就，市十一届人大四次会议通过的《南通市国民经济和社会发展第十个五年计划纲要》确定的主要发展目标全面超额完成。

　　--经济总量五年翻番，跨越发展态势强劲。全市地区生产总值按可比价年递增13.1%，财政总收入年递增21.7%。在多年蓄势的基础上，三年迈出三大步：2003年地区生产总值超千亿、财政收入超百亿；2004年地区生产总值增速超全省平均水平；2005年经济增长速度全省领先，地区生产总值、人均地区生产总值均比"九五"末翻了一番，财政总收入为"九五"末的3.2倍。农业综合生产能力稳步提高；二、三产业增加值占全市地区生产总值比重达89%，比"九五"末提高了6.9个百分点；2005年工业经济效益综合指数比"九五"末提高68个点。综合实力显着增强，在长三角城市中的位次不断前移。

　　--改革保持领先地位，开放型经济重返全省第一方阵。国有经济布局战略性调整和国有企业战略性改组目标基本实现，多种所有制经济竞相发展格局基本形成，市场化改革走在全省前列。争创江苏民营经济第一大市成效显着，主要指标增幅连续两年保持全省领先。行政审批事项大幅削减，政府服务效率明显提高；事业单位分类改革取得实效。实际利用外资持续实现"撑杆跳"，按原口径五年累计达到48.5亿美元，是"九五"的2.3倍；2005年进出口总额是"九五"末的2.7倍；外经合作保持省内领先。

　　--发展优势更加凸显，城市综合竞争力和影响力迅速提升。以苏通大桥开工建设、高速公路纵向横向贯通、铁路客货运通车、国道省道全面网化为标志的大交通格局初步形成。沿江开发强势推进，一批重大项目在沿江地区集聚落户，优势特色支柱产业快速扩张升级，沿江先进制造业和基础工业走廊基本形成；港口建设步伐加快，五年新增5万吨级以上泊位14座，2005年港口吞吐量是"九五"末的3倍。沿海开发实现重大突破，洋口、吕四海港开发建设实质性启动，南通开始从江河时代向江海时代跨越。发展后劲显着增强，全社会固定资产投资五年累计达2437亿元，是"九五"的2.2倍。连续14年实现耕地占补平衡。中心城市面貌变化巨大，"中国近代第一城"品牌彰显魅力；各县（市）城及重点镇面貌显着改观，城镇化进程明显加快，全市城市化水平比"九五"末提高了12个百分点。争创全省最佳办事环境，获中国城市管理进步奖，被评为最具台商投资价值城市。

　　--三个文明建设同步推进，经济社会协调发展。精神文明"南通现象"发扬光大，全国文明单位总数在全省名列前茅，建成江苏江北第一个文明城市群。民主法制建设进一步加强，依法治市五年规划目标和"四五"普法任务全面完成，政府工作法制化进程加快，基层民主法治示范村（社区）建设走在全省前列；人民群众对社会治安的满意率不断提高，被省委、省政府命名为2003―2005年度社会治安安全市，成为全省首批平安城市。社会事业全面发展，国民教育体系进一步完善，南通大学组建成立。群众文化生活不断丰富。全民健身体系加快健全。人口和计划生育工作进一步加强，人口连续8年负增长。老龄工作、社会福利等事业有较大发展。各类事故死亡人数连续5年下降。

　　--生活质量显着改善，人民群众得到更多实惠。城乡一体化的就业体制初步建立，五年累计提供就业岗位70万个，实现再就业19万人次，城镇登记失业率低于全国、全省平均水平。农村劳动力转移总量达到202.01万人，在全省领先。城镇社会保障体系基本形成。城乡居民收入实现较快增长，2005年市区居民人均可支配收入、农民人均纯收入分别是"九五"末的1.6倍和1.5倍。2005年末城乡居民储蓄存款余额1190.74亿元，是"九五"末的2.2倍。2005年城镇、农村人均住房建筑面积分别达到29.14和49.62平方米，比"九五"末分别增加12.3和8.17平方米。完成了城乡电网改造和通如区域供水工程，农村自来水基本普及。

　　各位代表，过去的五年，是我市发展史上不平凡的五年。在这五年中，我们主动适应宏观经济环境的新变化，积极应对加入世贸组织后的新形势，成功战胜非典和重大自然灾害的新挑战，不断把南通跨越发展大业推向前进。发展历程令人难忘，发展成就令人振奋。所有这一切，是省委、省政府和市委正确领导的结果，是市人大、市政协监督支持的结果，是全市人民团结拼搏、艰苦奋斗的结果，也是与历届领导创下的基础分不开的。在此，我谨代表市政府向广大干部群众，向人大代表、政协委员和离退休老同志，向人民解放军、武警驻通部队官兵和公安干警，向各民主党派、工商联、各人民团体和各界人士，向支持南通现代化建设的部、省驻通单位，向所有关心南通发展的港澳台同胞、海外侨胞和国际友人，表示崇高的敬意和衷心的感谢！

　　回顾过去，我们也清醒看到，全市经济社会发展中还存在很多矛盾和问题，主要是：与先进地区相比，在经济总量和均量指标上的差距还比较大；经济增长方式由粗放型向集约型转变的任务还很艰巨；自主创新能力不强，产业层次和集中度不高，重点企业支撑作用不够；消费需求对经济增长的拉动不足；城乡居民收入增加与经济增长不尽协调，农民持续增收、就业持续增加的难度加大，社会弱势群体生活困难还较多；统筹城乡、区域、经济社会协调发展和维护社会稳定的任务还很繁重。政府工作还存在缺点和不足，政府社会管理和公共服务职能仍需进一步加强等等。对此，我们将采取切实措施，认真加以解决。

　　二、坚持以科学发展观统领"十一五"经济社会发展全局

　　经过"十五"的发展，南通已经站在新的历史起点上。展望未来，"十一五"时期正是我市全面建设小康社会的决胜期，持续跨越发展的黄金期，提升在全省和长三角城市中地位的赶超期，经济社会发展进入全面腾飞的关键期。基于对发展态势的分析，市政府根据市委九届七次全会通过的《关于制定南通市国民经济和社会发展第十一个五年规划的建议》，在深入调查研究、广泛听取意见、集中各方智慧的基础上，编制了《南通市国民经济和社会发展第十一个五年规划纲要（草案）》（以下简称《纲要》），提出了"十一五"期间全市经济社会发展的总体思路、总体定位和主要目标任务。

　　《纲要》明确，"十一五"期间，要以邓小平理论和"三个代表"重要思想为指导，以科学发展观统领发展全局，根据省委、省政府作出的"新发优势在苏中"的科学判断，紧紧围绕经济社会全面腾飞的战略定位，继续按照"依托江海、崛起苏中、融入苏南、接轨上海、走向世界、全面小康"的发展思路，以建设"经济发达、文化繁荣、政治清明、社会和谐、人民安康"的新南通为战略目标取向，确保2010年、力争2009年在江苏江北率先全面建成小康社会，全面融入苏南板块，为在下个十年内基本实现现代化奠定坚实的基础，加快推动形成长三角核心区沪苏通"金三角"和沿海沪甬通"一体两翼"发展新格局，加速把我市建设成为江苏江海交汇的现代化国际港口城市、长三角北翼的经济中心和国内一流的宜居创业城市。

　　《纲要》提出，通过"十一五"的努力，做到：

　　--经济增长走在江苏前列。全市地区生产总值（可比价）年递增14%左右，2010年地区生产总值总量和均量比2005年翻一番。地方一般预算收入年递增20%以上，2010年力争超过200亿元。全社会固定资产投资累计超过6000亿元。注册外资实际到帐累计力争超100亿美元。2010年民营经济增加值占地区生产总值的比重达到60%。全市经济总量在全国城市排名中进入前30位。

　　--科学发展走在江苏前列。产业结构全面优化，经济增长方式实现较大转变，资源利用效率不断提高。2010年，二、三产业增加值占地区生产总值比重达到94%左右；单位生产总值能耗比"十五"末降低20%左右；全社会研发经费支出占地区生产总值的比重达到1.65%，市区达到2%左右；高新技术产业产值占规模以上工业产值比重力争超过25%。社会事业全面发展，科技、教育、人才的支撑作用明显增强，生态和人居环境有较大改善。

　　--和谐社会建设走在江苏前列。城乡、区域、社会群体之间收入分配差距扩大趋势逐步缓解。2010年，城镇居民人均可支配收入、农民人均纯收入分别超过20000元和8500元，居民文教娱乐服务支出占家庭消费支出比重超过20%，恩格尔系数小于40%；就业相对充分，城镇登记失业率控制在4%以内；社会保障体系和公共服务体系更加健全，卫生服务体系健全率达到96%，城镇养老、医疗、失业保险和新型农村合作医疗基本实现全覆盖，社会救助体系比较完备。民主法制建设和精神文明建设全面加强，社会治安居于全国先进水平。

　　为实现上述目标，《纲要》提出了"十一五"期间的八大发展任务，其核心是"三个坚持"：

　　（一）坚持跨越发展。围绕全面建设小康社会目标，始终把跨越发展作为主题，实现又快又好发展。

　　牢牢抓住机遇。"十一五"时期，将是南通期盼多年的诸多历史机遇集中迸发的时期。全省沿江沿海开发战略的实施，将使南通沿江优势进一步放大、沿海优势强劲凸现。苏通大桥及崇启、崇海大通道的建成，沪通铁路大动脉的建设，将使南通全面融入上海一小时都市圈，构筑起承南启北、西进东出，江海河联运、水陆空互通的"黄金十字通道"，在长三角经济一体化进程中获得发展先机。新一轮国际产业和资本加速向中国东部沿海转移，南通的生产要素比较优势也将在新一轮竞争中成为新发之机。为此，必须抢抓机遇、乘势而上，使"十一五"发展更有作为。

　　整合发挥优势。南通具有得天独厚的江海资源优势，具有紧邻上海、连通苏北的区位优势，具有特色鲜明的产业基础优势，具有政通人和、心齐气顺、崇尚创业、勇争一流的环境人文优势，只要我们注重挖掘、整合放大、深度利用，必将转化为巨大的综合竞争优势和推动跨越发展的现实生产力。

　　增强发展动力。始终坚持经济市场化、国际化发展方向，更大程度地发挥市场机制的作用，进一步提高对外开放水平，进一步激发全社会创造活力，为科学发展提供体制保障，为跨越发展提供强劲动力。

　　强化战略支撑。按市委九届七次全会要求，重点发展集约型、集群型、开放型、内生型、港口型和都市型等六大经济战略形态，抓好大通道、大港口、大物流、大能源、大市区、大企业、大项目、大县（市）等八大战略支点，为全面腾飞提供强力支撑。

　　（二）坚持科学发展。以集约、统筹、创新、节约为着力点，推动经济社会发展切实转入科学发展的轨道。

　　加快推进新型工业化。调整优化产业结构，加快形成以高新技术产业和优势产业为主导、先进制造业和基础产业为主体、现代服务业为支撑的新型产业格局。深入实施以工兴市第一方略，重点发展船舶制造与配套、现代纺织服装、机械电子、精细化工及新兴石油化工、轻工食品、电力能源等产业，加快培育行业领军企业，带动形成优势企业群，将南通建设成为长三角北翼先进制造业和基础产业基地。大力发展现代物流、金融保险、商务服务、信息服务、旅游等现代服务业，改造提升传统服务业，促进服务业发展的全面提速，实现服务业和制造业的互动发展。进一步打造"南通铁军"品牌，向全国一流建筑强市跨越。

　　加快新农村建设和城镇化进程。按照"生产发展、生活宽裕、乡风文明、村容整洁、管理民主"的要求，以工业化致富农民，以城市化带动农村，以产业化提升农业，千方百计增加农民收入，扩大公共财政覆盖农村的范围，扎实建设"民富、村美、风气好"的社会主义新农村。实施积极的城市化战略，大力推进中心城市建设，加快发展中小城市，培育重点中心镇，构建层级分明、功能互补的城镇体系，城市化水平超过55%，形成市区经济与县（市）经济互补共进的发展格局。

　　加快建设创新型城市。坚持科教兴市、人才强市战略，为建设创新型城市提供强有力的科教人才支撑。着力提高自主创新能力，加大科技投入，培育科技创新主体，促进企业和产业核心竞争力的全面提升，力争建成全国科技进步示范市和国家知识产权示范城市。优先发展教育，重视发展义务教育、高等教育，加速发展职业教育，推动教育均衡发展，逐步建立开放式、多层次的终身教育体系。营造人才发展环境，构筑南通人才高地。

　　加快建设资源节约型和环境友好型社会。积极发展循环经济，提高资源循环利用水平。以建设节约型机关推动节约型社会建设。坚持保护与建设并举，源头控制与末端治理并重，实行最严格的环境保护制度，有效规避产业转移带来的环境风险，建成国家环保模范城市群。以积极的环境建设改善自然生态。

　　（三）坚持和谐发展。关注民生，维护民权，妥善处理不同利益群体关系，使全市人民共享改革发展成果，构建和谐南通。

　　坚持富民优先。学习昆山经验，大力倡导和支持全民创业、自主创业，提高资产性、经营性收入的比重，多渠道增加城乡居民收入。强化政府就业服务，完善市场调节就业机制，千方百计实现相对充分就业。围绕人人享有社会保障的目标，构筑广覆盖的社会保障安全网，提高养老、医疗、失业等社会保障水平。全面建立被征地农民基本生活保障制度。

　　注重维护公平。综合运用多种手段，保障和促进社会公平。构建和谐劳动关系，合理调节收入分配，着力提高低收入者收入水平，逐步扩大中等收入者比重，努力缓解收入差距扩大的趋势。注重帮扶弱势群体，建立新型社会救助、优抚安置、社会福利等帮扶社会弱势群体的公共事务管理体系。加快构建以权利公平、机会公平、规则公平、分配公平为主要内容的社会公平保障机制，使人民群众平等享有政治、经济、社会、文化权利。

　　加强社会建设和管理。完善社会管理和公共服务体系。加快发展文化事业和产业，努力建设文化大市、体育强市。健全新型公共卫生和医疗服务体系。加强人口和计划生育工作。大力发展老龄事业和产业，强化妇女、儿童、青少年、残疾人等工作。加强精神文明建设和民主法制建设，大力弘扬"包容会通、敢为人先"的新时期南通精神。深入推进法治南通、平安南通、诚信南通建设，全面落实安全生产、社会治安各项措施，努力形成安定、安全、安宁的良好局面。

　　三、为"十一五"发展起好步开好局

　　2006年是"十一五"的开局年。政府各项工作必须在高平台上实现高起步、新起点上实现新突破，全面开创跨越发展、科学发展、和谐发展新局面，确保"十一五"首战必胜。

　　今年全市经济社会发展的主要预期目标是：地区生产总值增长14%以上，单位生产总值能耗下降4%，全社会固定资产投资增长25%以上，注册外资实际到帐额增长30%，社会消费品零售总额增长15%，地方一般预算收入增长18%以上，城镇居民人均可支配收入、农民人均纯收入分别增长11%、10%，城镇登记失业率控制在4%以内，居民消费价格指数涨幅控制在3%左右，人口自然增长率控制在零以下。

　　实现上述目标，将在以下十个方面取得新突破：

　　（一）着力争先进位，实现工业经济发展新突破。坚持以工兴市第一方略，确保规模工业增加值增长18%以上。全力做强做大企业。支持大企业加快资本扩张和技术创新，力争全市销售收入超亿元工业企业达500家，其中超10亿元企业达30家。全面实施"微小企业上规模"工程，培育新进规模工业企业1000家，总数超过4000家。健全国有资产监管体系，进一步做好企业改制的后续完善工作，加快上市公司股权分置改革。持续加大有效投入。着力抓好总投资超千万元的574个结转项目和520个新上项目，确保工业投入超720亿元。加速转变增长方式。用先进技术改造传统产业，更新落后工艺和装备，提高资源利用率和原材料转化率。加快发展高新技术产业，力争产值超过500亿元；实施自主创新能力提升"112"工程、知识产权"315"工程、产学研合作"百千万"工程，重点推进100个自主创新和产学研项目。启动实施循环经济"1668行动计划"，大力推进资源节约和综合利用。加快土地等后备资源和风能等可再生能源开发。按照"集合要素、集约开发、集中布局、集聚发展"的要求，引导工业加速向省级以上开发区、重点工业集中区集中，提高土地利用率、建筑容积率和投入产出率。按计划推进市区三类企业搬迁改造和"三镇两行业"污染治理、"四镇两流域"水系治理工作，制定实施污染物总量控制和削减计划，全面推行排污许可制。

　　（二）坚持城乡、区域协调发展，实现新农村建设新突破。以"民富、村美、风气好"为目标，学习华西等先进典型，扎实推进社会主义新农村建设。以农民收入倍增工程为抓手，构筑农村新产业。稳定粮食生产。面向市场调整结构，继续采取项目推进的办法，大力发展高效型、外向型、都市型农业，力争农业增加值增长4%。加快构建优势农产品特色产业带，促进高效农业规模化，力争中高效农田面积占比提高5个百分点。搞好国土整理、复垦、开发，继续实现耕地占补平衡。切实改善农业生产条件，提高农业机械化水平。鼓励、支持村干部、农村能人创业致富，带富一方。大力发展劳务经济，增加农民非农收入。积极发展各种新型社会化服务组织。开展村级集体资产股份合作制改革试点，加强农民负担监管和农村财务管理，积极化解镇村不良债务，规范村级"一事一议"筹资筹劳。以科学规划为龙头，打造农村新环境。坚持"规划先行、分步实施、建管并举"，推进农村空间布局规划全覆盖，初步完成县（市）级土地利用总体规划修编工作。深入实施"百村示范、千村整治"工程，大力开展以建立健全农村环卫体系为重点的农村环境整治活动。继续加快交通、水利、管网等基础设施和基础教育、基层文化、基本医疗设施建设，扎实稳步开展农民集居区建设。加快发展循环农业。着力推进沿江、沿海生态防护林和农田林网等工程建设，确保森林覆盖率增加0.8个百分点。以基层组织建设为支撑，培养造就新农民。关心广大基层干部，以先进性教育为契机，提高基层执政能力和管理服务水平。继续推进村务公开、民主理财，完善基层民主选举制度。广泛开展农村精神文明创建活动，建立健全村规民约，加强从业技能培训，提高农民整体素质。促进区域经济竞相发展，构建区域协调发展新格局。切实加强分类指导，巩固提升区域均衡发展良好态势，推动县（市）区在全省位次前移，促进北三县（市）继续保持强劲发展势头，南三县（市）争当率先融入苏南排头兵，三区增强对全市经济发展的辐射带动力。

　　（三）加快发展现代服务业，实现服务业发展新突破。继续把服务业放在优先发展的位置，力争提高服务业在地区生产总值中的比重。突出发展现代物流业。重点推进大型港口物流园区、铁路东站物流中心和海安物流中心等"一园区、五中心"建设，重点培育南通港口集团等10家现代物流企业，大力推进空港建设，注重培育第三方、第四方物流。加快发展商务服务业。吸引外地金融机构来通设立分支机构，积极引导民间金融和地方金融机构发展。大力发展资本、产权、技术等要素市场和法律、会计、设计、咨询、会展等服务组织。不断拓展信息、软件、网络服务业，力争"南通软件园"建设有新突破。提升发展现代商贸业。在提升市区老城区商贸业发展层次的同时，加快新区商务区建设，打造新的都市商圈。创新商贸形态，优化网点布局。做大做强专业市场，确保年成交额超10亿元的市场达9个。保持房地产业健康发展。加快工业、商业房产开发建设，继续推动商品住宅规模开发、成片改造，搞活房地产二级市场。大力发展特色旅游业。启动"旅游项目建设三年行动计划"，加快建设沿江、沿海旅游带和大桥景区等一批旅游项目，力争旅游总收入突破90亿元。举办中国旅游文学论坛暨首届"徐霞客旅游文学奖"颁奖活动。完善旅游基础设施，规划建设一批高档旅游饭店。

　　（四）巩固提升全省第一方阵位次和水平，实现开放型经济发展新突破。坚持量质并举，推动利用外资新跨越。大力引进船舶制造与配套、石油化工、物流仓储等临港产业项目，纺织服装、机械制造、轻工食品等优势产业项目，电子信息、生物医药等高新技术产业项目。重点跟踪推动王子制纸增资等10个单体投资规模超亿美元的重特大项目。积极引导外资投向现代服务业。强化招商攻势，精心组织好港口经济洽谈会等重大活动和产业专题招商，加强与外国使领馆和跨国公司的联系。完善招商引资推进机制，提高外资到帐率、项目落地率、开工投产率。加快结构调整，拓展外贸发展新空间。优化出口商品结构，扩大自主知识产权、自主品牌产品出口。积极探索贸易方式多元化，大力发展技术贸易和服务贸易。积极应对贸易磨擦。提高"大通关"效率。力争出口总额增长20%。提升合作层次，促进外经跃上新台阶。支持优势企业参与国际工程承包，完善外经合作预警、自律、协调机制和纠纷处理快速反应机制，确保对外承包劳务营业额增长10%以上。鼓励有条件的企业充分利用国（境）外资源、市场，加快"走出去"步伐。加强载体建设，营造开发区发展新优势。发挥好国家级开发区龙头作用，大力提升南通出口加工区功能，推动全市省级以上开发区争先进位，国家级开发区在全省省级以上开发区中的位次力争前移两位。

　　（五）大力实施"三名"工程，实现民营经济发展新突破。继续围绕争创江苏民营经济第一大市的目标定位，以推进"名企·名品·名人"工程为抓手，着力培育规模优势、质量优势和竞争优势。继续推动全民创业。大力弘扬"三名"典型的创业创新创优精神，进一步在全社会营造浓烈的创业氛围，激发更多民众投身创业，保持全民创业强劲势头。大力提升发展层次。制定并实施"十一五"争创名品、名企规划，力争今年每县（市）区创建1件以上中国名牌产品或驰名商标、3件以上江苏名牌产品或着名商标。加强对重点民营企业争创名牌、信贷担保、人才培训、境内外上市等全方位服务，促进重点企业快速扩张。加速做大特色产业。重点培育22个特色经济板块。着力发挥重点龙头企业的辐射带动作用，促进产品前延后伸，拉长产业链，推动区域特色产业发展壮大，每个特色产业重点培育3家以上亿元龙头企业。以大企业（集团）为核心，推进专业化分工，培植一批中小企业群。围绕提高核心竞争力，制定并实施建设全国一流建筑强市的政策意见，切实强化引导扶持，力争完成建筑业施工产值900亿元。

　　（六）猛攻重特大项目，实现江海联动开发新突破。纵深推进沿江开发，抓紧研究并组织实施加速推进沿海开发的举措，以项目带动开发，增创江海联动发展新优势。全力突破产业大项目。着力抓好中远川崎二期、熔盛造船、醋酸纤维、江苏LNG等100个重点项目建设，重点跟踪推进单体投资规模超5亿元的44个重特大项目，抓紧做好50个重大产业储备项目的前期工作，确保全社会固定资产投资突破1000亿元，其中规模以上投资占比达85%。重点建设江海大港口。深入推进沿江岸线整合、整治、开发，继续做好新通海沙、新开沙夹槽、横港沙、泓北沙和长江北支等整治开发前期工作。加速推进洋口港陆岛通道、洋口大道、人工岛等工程建设，启动吕四港规模开发前期论证工作。提升沿江沿海港区功能，实现亿吨大港目标。加快构筑立体大交通。进一步加强苏通大桥建设服务工作，开工建设崇启大桥，力争崇海大桥年内动工。加快沿江高等级公路建设，做好扬启高速海安段开工准备，推进204国道、334省道改造工程。开工建设铁路南通站、南通站至南通东铁路和狼山港铁路支线，力争开工建设沪通铁路，积极做好洋口港铁路和江海港区、如皋港区铁路支线前期工作。积极参与区域大合作。加快与上海全面接轨。组织推进长三角城市合作专题，加速融入长三角经济一体化进程。

　　（七）以承办省十六运为契机，实现城市建设新突破。进一步加大城市建设力度，为省十六运等重大活动创造一流环境条件。进一步提升城市形象。按照"江海特色的现代版、文化浓缩的精品区"的新区定位和快出形象的要求，按期建成体育会展中心，加快推进中央商务区建设；以启动园博园建设为契机，全面加快五山景区和周边区域整体开发建设步伐。推动老城区改造提升，继续做优做精濠河景区，加快八仙城二期等环濠河改造重点工程的拆迁和建设进程。启动火车站站前区开发，建成城闸大桥，搞好唐闸、天生港等4个片区规划建设，在港闸区加快建设区域性商贸中心。进一步完善城市功能。突出解决市区交通难问题，继续优化城市路网结构，新建和改造20条城市主次干道、20条重要支路和滨江大桥、通吕四号桥，基本形成"三环六射"道路网络；抓紧解决南大街商圈交通拥堵问题，提高市区核心区域通行能力。继续加强城区污水排放、处理和绿化、停车、公交、电力等基础设施建设。积极探索城市中水回收利用。完善县（市）城建设规划，强化产业支撑功能，促进人口集聚，加快向中等城市发展。进一步推进"五城同创"。强化城市综合执法，完善城市长效管理机制，提升城市管理水平。继续推动老小区封闭管理试点。巩固国家环保模范城市创建成果，推进环保模范城市群建设，启动生态市建设；争取成为国家卫生城市；完成国家园林城市创建申报准备工作；积极申报省级历史文化名城。

　　（八）加快科教兴市、人才强市进程，实现社会事业发展新突破。大力发展科技事业。继续加强科技孵化器建设，启动南通科技创业社区建设。加快发展技术产权、科技咨询等科技服务组织，提高科技社会化服务水平。进一步完善科技进步考核体系。加强科普宣传，提高全民科学素质。优先发展教育事业。巩固义务教育"双高普九"成果，确保农村中小学创建合格学校工作通过省级验收。加强学前教育和成人教育，大力发展职业教育，继续支持南通大学加快发展。强化人才队伍建设。积极推进省级人才特区试点，加强人才载体和环境建设，突出创新型、技能型、应用型人才的培养和引进，完善人才激励机制，促进人尽其才、才尽其用。提高全民健康水平。完善新型公共卫生体系，健全城乡社区卫生服务网络。启动"农民健康工程"，完善城乡全民健身体系。继续稳定低生育水平，提高出生人口素质。精心承办好省十六运。集中各方力量，整合各类资源，制订各种预案，高标准做好参赛准备，高水平抓好竞赛组织，从严从细做好开幕式等重大活动的筹备工作，以成功承办省十六运充分展示新时期南通形象。加强精神文明建设，加快建设文化大市。全面推进各类群众性精神文明创建活动，大力弘扬南通精神。加强未成年人思想道德建设。继续光大精神文明"南通现象"。积极推进"诚信南通"建设。加强"中国近代第一城"文化遗产保护利用，打造"青墩遗址"文化品牌。强化基层文化阵地建设。加快发展文化产业。积极发展广播电视和新闻出版事业。繁荣哲学社会科学。进一步加强国防后备力量建设，支持驻通部队建设，继续创建全国双拥模范城。继续加强统计、人防、档案、地方志、保密、气象、防震减灾、无线电管理等工作，做好民族、宗教和侨务、对台工作。

　　（九）切实解决人民群众最关心、最直接、最现实的利益问题，实现和谐社会建设新突破。着力做好就业再就业工作。进一步落实就业和再就业政策，健全完善就业服务体系，加强就业再就业援助工作，支持自主创业、灵活就业、自谋职业。加强对用工单位工资收入分配的指导和调节，积极推进企业工资协商机制建设，维护广大职工和农民工合法权益。不断完善社会保障体系。切实推进城镇企业职工社会保险全覆盖。加快推进城乡养老保险一体化，健全多层次的医疗保障体系，提高工伤、失业、生育保险覆盖面。完善农村五保户制度和被征地农民基本生活保障制度。切实帮助城乡低收入群众解决住房、医疗、子女就学等困难。加强救灾救济和社会福利、社会救助等工作。积极发展老年人和残疾人事业，切实保障妇女儿童合法权益，加强青少年保护。继续做好扶贫和老区开发工作。全力维护社会稳定。加强法制宣传教育和基层民主法治基础工作，开展法治合格县（市）区、乡镇和单位创建活动。健全大信访工作体系，完善处置群体性事件工作机制。巩固社会矛盾纠纷大调解机制和社会治安大防控格局，建立严打整治长效机制，提升平安南通建设水平。严格执行《安全生产法》，增加安全生产投入，加强职工安全知识培训，落实安全生产责任制，加大安全生产监管力度，坚决遏制重特大事故发生。进一步整顿和规范市场经济秩序，严厉打击商业欺诈，切实抓好食品安全、药品市场、农资打假、非法采供血等专项整治。继续办好惠及群众的实事工程。(1)统筹城乡就业，提供就业岗位12万个，其中市区4万个；免费开展农村劳动力转移培训6万人、农业实用技术培训1万人、创业培训4000人。(2)巩固新型农村合作医疗，覆盖面达85%。(3)对农村义务教育阶段学生免收杂费，对城乡义务教育阶段贫困家庭学生实行"两免一补"。(4)围绕2007年建成的目标，加快西北片区域供水建设进度，完成启海片区域供水一期工程。(5)加强农村环境综合整治，全面实施"三清"工程，疏浚市级河道2条段、县级河道18条段、乡级河道180条，清淤土方2200万方；集中整治市区河道12条段。(6)改、扩建农村桥梁500座，新建和改造农村公路350公里，实现村村通水泥路、沥青路。(7)建设市区老年活动中心，改善市老年大学办学条件，市区新增社区老年活动场所10个。(8)建设市区残疾人综合服务中心，向3000名盲人捐赠盲杖，向1000名下肢残疾人捐赠轮椅车。(9)市区优化公交线路10条，新增和改造公交站台（点）50个，切实改善城市公交乘车候车条件。(10)对市区3个老住宅片区实施以雨污分流、水系贯通为重点的综合改造；市区新建低价位商品房60万平方米；安排3000万元为市区住房困难家庭提供政策性住房保障。(11)市区新建农贸市场2个，改造提升4个。(12)市区人民公园、文峰公园、唐闸公园、滨江公园免费向市民开放。

　　（十）努力构建服务型政府，实现发展软环境建设新突破。按照"为民、务实、清廉"的要求，加快建设服务型政府、责任政府、法治政府和廉洁政府。推进管理创新。更加注重履行社会管理和公共服务职能，建立健全自然灾害、事故灾难、公共卫生、社会安全等突发公共事件预警和应急处理机制，认真抓好重大动物疫病防治工作；更加注重体制机制创新，进一步推进政企、政资、政事、政府与中介组织分开，基本完成市属社会公益型事业单位改革，加快县乡财政管理体制和乡镇机构改革；更加注重提高服务效能，继续以"争创全省最佳办事环境"为目标，加强行政效能建设，激励多干事、干成事，惩戒乱作为、不作为，为人民群众和投资者创造良好的服务环境，为企业创造公平的竞争环境。提高落实成效。把"十一五"发展的目标任务落实到具体部门、具体项目和具体工作上；把"十一五"首战必胜的要求落实在调查研究、基础工作的加强上，落实在要素等瓶颈制约、拆迁等难点问题的化解上，落实在重大项目、重点工作的突破上。完善决策机制。认真开展决策调研，完善社会公示、重大决策听证和专家咨询制度，提高决策的民主化、科学化、规范化水平。对与群众利益密切相关的重大决策，认真听取人大代表、政协委员的意见，通过多种渠道、多种形式，广泛征询社会各界人士的建议。坚持依法行政。把依法行政作为政府工作的基本准则，全面启动法治南通建设工程。推进综合执法改革，规范行政执法行为，继续做好行政复议工作，严格实施行政执法责任制和过错追究制。认真贯彻人大及其常委会的决议、决定，及时主动报告工作，自觉接受法律监督和工作监督；自觉接受政协的民主监督，加强与各民主党派、工商联和各人民团体的联系与沟通。高度重视办好人大议案和代表建议、批评、意见及政协委员提案。强化行政监督。进一步拓宽政府信息公开范围，依法保障公民的知情权、参与权和监督权。加大执法监察、效能监察、廉政监察和财政资金使用监管的力度，强化重点领域、重点部门、重点项目审计。加强队伍建设。全面落实党风廉政建设责任制，建立健全教育、制度、监督并重的惩治和预防腐败体系，严肃查处损害群众利益的突出问题和违纪违法案件。认真贯彻落实《公务员法》，建设一支善于学习、忠于职守、清正廉洁、作风优良的公务员队伍。

　　各位代表，南通全面腾飞的序幕已经拉开，新的形势催人奋进，新的任务光荣艰巨。让我们在省委、省政府和市委的正确领导下，高举邓小平理论和"三个代表"重要思想伟大旗帜，全面落实科学发展观，解放思想、开拓创新、求真务实，为实现跨越发展、科学发展、和谐发展宏伟大业而努力奋斗！
